# The Waelz Slag from Electric Arc Furnace Dust Processing: Characterization and Magnetic Separation Studies

**DOI:** 10.3390/ma17102224

**Published:** 2024-05-08

**Authors:** Pavel Grudinsky, Anfisa Yurtaeva, Denis Pankratov, Liliya Pasechnik, Roman Musaelyan, Valery Dyubanov

**Affiliations:** 1 I.P. Bardin Laboratory of Issues of Complex Ore Metallurgy, A.A. Baikov Institute of Metallurgy and Materials Science, Russian Academy of Science, 49 Leninsky Prosp, 119334 Moscow, Russia; anfisayurtaeva@mail.ru (A.Y.); vdyubanov@imet.ac.ru (V.D.); 2 Faculty of Biotechnology and Industrial Ecology, D. Mendeleev University of Chemical Technology of Russia, 9 Miusskaya Square, 125047 Moscow, Russia; 3 Department of Radiochemistry, Faculty of Chemistry, Lomonosov Moscow State University, 1-3 Leninskiye Gory, 119991 Moscow, Russia; pankratov@radio.chem.msu.ru; 4 Institute of Solid State Chemistry, Ural Branch of the Russian Academy of Sciences, 91 Pervomaiskaya St., 620990 Ekaterinburg, Russia; pasechnik@ihim.uran.ru; 5 FRC V.V. Dokuchaev Soil Science Institute, 7 bdg. 2 Pyzhevsky Lane, 119017 Moscow, Russia; romaniero1@gmail.com

**Keywords:** Waelz slag, electric arc furnace dust, magnetic separation, zinc, copper, iron, Mössbauer spectroscopy

## Abstract

The Waelz slag generated during electric arc furnace dust processing is an iron-rich product with significant amounts of iron, zinc and copper. About 600–800 kg of the Waelz slag is generated per ton of the dust processed. The Waelz slag samples from two different plants were thoroughly characterized using inductively coupled plasma optical emission spectroscopy (ICP-AES), X-ray diffraction analysis (XRD), chemical phase analysis, Mössbauer spectroscopy and other supporting methods. The phase distribution of iron, zinc and copper was determined in the Waelz slag samples. Low-intensity wet magnetic separation was tested for the iron recovery from the Waelz slag samples. It was found that the Waelz slag samples have complex chemical and mineralogical compositions, which can impede the selective recovery of valuable elements. The obtained results indicate that the chemical and mineralogical composition of the Waelz slag samples has a considerable effect on the magnetic separation indexes. The experiments showed that the iron concentrates with Fe contents of 73% and 46.8% with the metallization degrees of 87.2% and 57.5% and the iron recovery degree of 54.8% and 52.9% were obtained at optimal conditions for two different samples, respectively, without selective segregation of Cu and Zn in the magnetic or non-magnetic fraction.

## 1. Introduction

In electric furnace steelmaking, steel is often made from galvanized steel scrap that leads to the production of a high-zinc by-product named electric arc furnace dust (EAFD). By various estimates, electric furnace steelmaking generates 5–8 million tons of EAFD in the world annually [1,2], and its considerable increase is predicted to be at least 18 million tons by 2050 [3]. The high zinc content in the EAFD, which can achieve more than 30% [4], makes it a valuable resource for zinc production. A combined percentage of zinc and iron can achieve more than half of the composition of the EAFD, but the zinc recovery is the main motivation due to its profitability. Zinc hydrometallurgical plants require at least 55% Zn [5,6] in raw materials for production, so the EAFD treatment is needed to separate zinc from other elements such as Fe, Ca, Mn, Cl, etc., and obtain the zinc-rich product. The most applicable method to derive the product enriched in zinc is the Waelz process [7], which has a market share of over 85% among the other available processes. It comprises the carbothermic reduction roasting using a rotary kiln at a temperature up to 1300 °C. Zinc volatilizes during the roasting, then oxidizes in a gas exhaust duct and collects in a chamber [8]. The obtained chamber product, named the Waelz oxide, is a valuable raw material for hydrometallurgical zinc production [9].

The Waelz process also produces another solid product named the Waelz slag, which contains the main part of the residual elements of the treated EAFD. It is estimated that the processing of every ton of EAFD generates 600–800 kg of the Waelz slag [10,11]. It is mostly not recycled but landfilled or stored on-site, thereby causing environmental risks [12]. There are some reports on the use of the Waelz slag in the construction industry [13,14] and road making [15,16]; however, high contents of toxic metals often impede meeting the requirements of leachability tests for building materials [17]. In order to prevent the leaching of toxic metals from the Waelz-slag-containing building materials, a preliminary stabilization treatment is needed [18,19]. Iron is the base component of the Waelz slag, but too-high zinc content hinders its utilization in the ironmaking process [20]. Few studies on pyrometallurgical methods for the Waelz slag treatment to remove zinc and lead have been carried out [21,22], but even after the treatment, the residue obtained was unsuitable for the application in iron and steel industry due to the high contents of the impurities. A recent study was conducted [23] on the magnetic separation of Waelz slag for iron recovery. This approach deserves attention because it may provide the segregation of valuable elements without the use of costly and environmentally hazardous pyrometallurgical or hydrometallurgical methods. Moreover, a valuable iron-rich product can be obtained from the Waelz slag.

In this paper, we identify the behavior of zinc, copper and iron during the Waelz slag processing using low-intensity wet magnetic separation. For this purpose, chemical and mineralogical composition, as well as other properties of the Waelz slag samples from two different plants, were characterized. Based on the obtained experimental results, the prospects and applicability of the Waelz slag processing to recover valuable elements from the Waelz slag were discussed.

## 2. Materials and Methods

### 2.1. The Waelz Slag and Its Products Characterization

The Waelz slag bulk samples were shipped from JSC “Chelyabinsk zinc plant” (Chelyabinsk, Chelyabinsk region, Russia, 55.210339° N, 61.376907° E) and “Alfatrans” LLC (Nizhny Tagil, Sverdlovsk region, Russia, 57.950634° N, 60.009374° E) and named WS1 and WS2, respectively. The sampling was carried out as follows: The reduction in the bulk samples of approximately 10 kg was performed using cone quartering to derive the intermediate samples of about 1 kg, which were used for characterization and magnetic separation studies. Analytical samples were obtained from the intermediate samples using sequential crushing in a cast-iron mortar, grinding in a ball micromill Fritsch Pulverisette 7 premium line (Fritsch, Germany), screening through a sieve with a mesh size of −0.054 mm, and reduction by quartering to about 50 g.

The chemical composition of the Waelz slag samples was analyzed by an inductively coupled plasma atomic emission spectrometer (ICP AES) Vista Pro (Varian Optical Spectroscopy Instr., Mulgrave, Australia). The contents of carbon and sulfur were measured by a LECO CS-230 gas analyzer (St. Joseph, MI, USA). The content of total iron was determined using the potassium dichromate titration method based on the standard [24] modified by the work [25] due to the high copper content in the samples. Potassium dichromate titration methods were also used to analyze the contents of ferrous [26] and metallic iron [27]. The content of ferric iron was calculated as a difference between the total and sum of metallic and ferrous iron.

The XRD patterns of the Waelz slag samples, as well as the samples after magnetic separation, were obtained by diffractometer SmartLab SE (Rigaku, Tokyo, Japan). The shooting mode of the XRD patterns was as follows: Cu-K_α_ radiation of 1.54178 Å wavelength, X-ray tube Toshiba (2.2 kW, 40 kV, 50 mA), graphite monochromator, angular speed of 2 2θ°/min with a step of 0.02°, Bragg–Brentano geometry. The holder for the samples was a cassette for 10 cuvettes. The samples were made by grinding to obtain powders with a particle size of less than 0.05 mm and applying undirected preparation to the surface of the low-background cuvette. The diffractometer was controlled by SmartLab Studio 4.5.397.0 software (Rigaku, Tokyo, Japan). The qualitative phase analysis was performed by Match! 3.15 Software (Crystal Impact, Bonn, Germany) [28] using Crystallography Open Database [29].

^57^Fe Mössbauer absorption spectra were measured in transmission geometry with a moving source and triangular velocity reference signal on an express Mössbauer spectrometer MS1104EM (CJSC Kordon, Rostov-on-Don, Russia) at temperatures of 296 ± 3 K or 77.7 ± 0.3 K. The source of γ-radiation in the form of ^57^Co in a matrix of metallic rhodium with an activity of 40 mCi (Cyclotron Co., Ltd., Obninsk, Russia) was at room temperature. The reference absorber of α-Fe foil was used for the velocity calibration. The noise/signal ratio did not exceed 2%. The mathematical processing of the experimental Mössbauer spectra was carried out for high-resolution spectra (1024 points) using the SpectRelax 2.8 software (Lomonosov Moscow State University, Moscow, Russia). The values of the isomeric shift are indicated relative to α-Fe at 296 K.

The quantitative phase analysis of zinc and copper minerals was carried out using chemical phase analysis [30]. Selective leaching of zinc sulfates, oxide, silicates and sulfide was carried out using water, ammonia chloride solution, acetic acid solution and hydrogen peroxide solution, respectively; our previous paper [31] describes the zinc analysis steps in detail. Sulfide and metallic copper were leached using a methanol bromine solution, then boiled with acetic acid to dissolve copper silicates, and finally, a residual part of sulfide and metallic copper, which was intergrown with silicates, was leached using the same methanol bromine solution. Appendix A includes a detailed schematic diagram of copper mineral analysis.

The Waelz slag samples of 15–40 mg in weight were analyzed by simultaneous thermogravimetric and differential scanning calorimetric method (TG–DSC) in an atmosphere of air or argon in corundum crucibles by SDT Q–600 (TA Instruments, New Castle, TE, USA) device with a heating rate of 20 °C/min in the temperature range of 25–1000 °C. The prepared polished sections of the samples were investigated by a scanning electron microscopy method using JSM 6390LA (JEOL, Tokyo, Japan) device equipped with an energy-dispersive X-ray (EDX) detector JED2300 (JEOL, Tokyo, Japan).

Sieve analysis was performed by a vibrating screener using 8 different screens with an aperture size of 10, 7, 2.5, 1.6, 1, 0.4, 0.2 and 0.1 mm for 1 kg of the Waelz slag samples. After the screening, if it was necessary, the appropriate fraction was ground to −0.4 mm, then a specimen of about 5 g was taken by quartering and analyzed iron, zinc and copper using the above-mentioned titration and ICP-AES methods.

Photomicrographs of the polished sections of the Waelz slag samples were obtained using METAM LV-34 (LOMO-Mikrosistemy, Saint Petersburg, Russia) optical microscope by reflected light.

### 2.2. Magnetic Separation

The Waelz slag samples were ground using the micromill Fritsch Pulverisette 7 premium line (Fritsch, Germany) or an agate mortar with a pestle, depending on the required fineness degree. Wet magnetic separation was realized by the XCGS-50 (Shaoxing Weibang Mining Machinery Manufacturing Co., Ltd., Shaoxing, China) device (Davis tube) with a magnetic field strength in the range of 0.5–4.5 kOe. The device consists of a glass tube with a diameter of 50 mm and a length of 770 mm, which is placed between the poles of the electromagnetic system. The Waelz slag sample of 10 g in weight of a required grinding fineness was placed into the device, which was filled with tap water with preset required magnetic field strength. The translational and rotational motion of the tube with a tap water flow led to the separation of the magnetic fraction, whereas the non-magnetic particles were poured out through a hose at the end of the tube into a container. After the tap water flow became clear, the electromagnetic system was turned off, and the magnetic fraction was poured out through the hose in another container. The obtained products were filtered using a suction flask and a porous porcelain filter, dried at 105 °C for 120 min and then weighted.

## 3. Results

### 3.1. Waelz Slag Characterization

#### 3.1.1. Chemical Analysis

Table 1 shows the chemical composition of the Waelz slag samples.

As can be seen from the analysis data, both samples have quite different chemical compositions. The WS1 sample involves substantial amounts of copper, arsenic and carbon, while the amounts in the WS2 sample are considerably less. In addition, it should be noted that the contents of calcium and zinc in the WS2 sample are more than twice that of the WS1 sample. The iron content in both samples is attractive for magnetic separation tests.

#### 3.1.2. XRD Analysis

**Figure 1:**
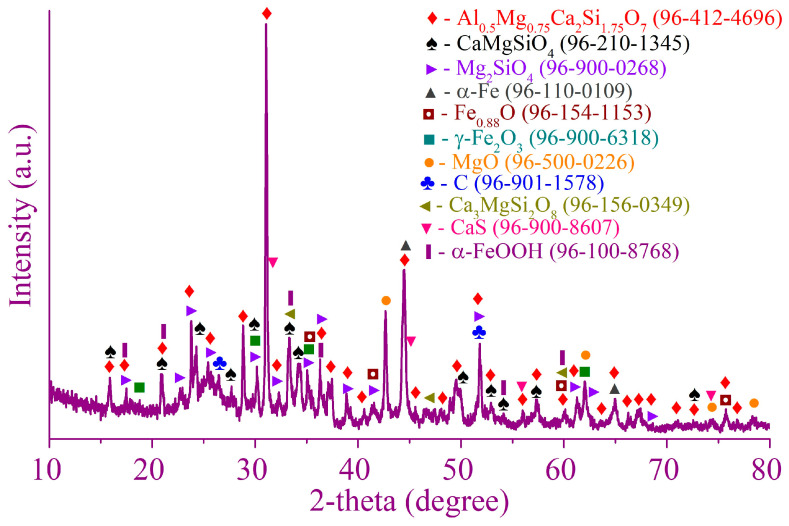
The XRD pattern of the WS1 sample.

**Figure 2:**
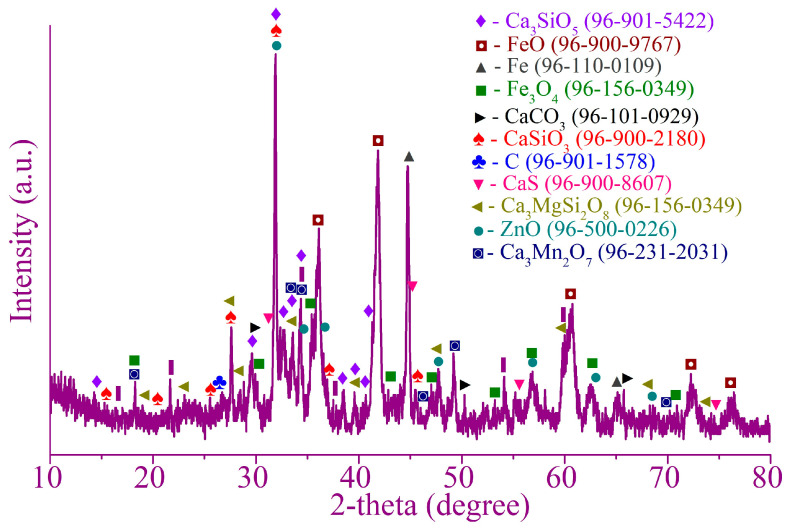
The XRD pattern of the WS2 sample.

Figure 1 and Figure 2 illustrate the XRD patterns of the Waelz slag samples WS1 and WS2, respectively.

As follows from the XRD patterns, the main identified iron-bearing minerals of both samples are metallic iron Fe, wustite Fe_1−x_O, magnetite Fe_3_O_4_ and goethite α-FeOOH. The iron-bearing phases have a different distribution in the samples; for example, the WS2 sample has strong reflexes of wustite, whereas they are much weaker in the WS1 sample. Carbon is mostly in the form of graphite, which is the residual coke breeze added for the Waelz process. The main iron-free phases of the WS1 sample are melilite Al_0_._5_Mg_0_._75_Ca_2_Si_1_._75_O_7_, monticellite CaMgSiO_4_ and forsterite Mg_2_SiO_4_, while in the WS2 sample, they are tricalcium silicate Ca_3_SiO_5_ and calcite CaCO_3_. A variety of the iron-free phases is due to a difference in the chemical composition of the samples (Table 1). In the WS2 sample, weak ZnO reflexes are observed, which correlates with the chemical composition given above.

#### 3.1.3. Thermal Analysis

**Figure 3:**
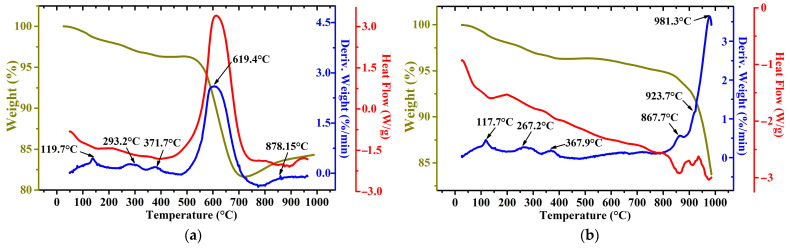
TG–DTG/DSC plots of WS1 sample in air (**a**) and argon (**b**) atmosphere.

**Figure 4:**
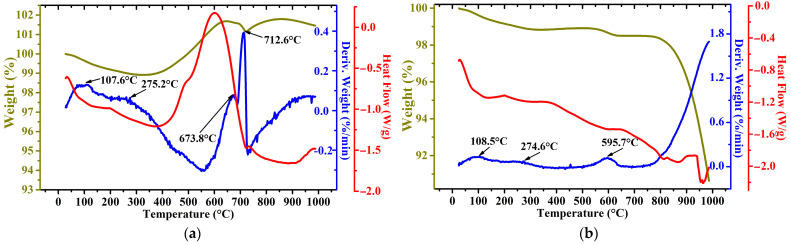
TG–DTG/DSC plots of WS2 sample in air (**a**) and argon (**b**) atmosphere.

Figure 3 and Figure 4 show TG–DTG/DSC curves obtained in the air and argon atmosphere of the WS1 and WS2 samples, respectively.

As can be seen from the obtained curves, the first peak for both samples, which is observed in the temperature range of 105–120 °C, is associated with the evaporation of free and inherent moisture. The origin of the exothermic peaks around 270 °C is likely due to the dehydroxylation of α-FeOOH to obtain hematite α-Fe_2_O_3_ [32]. The peaks for the WS1 sample at 371 and 368 °C in an atmosphere of air and argon, respectively, can be explained by the beginning of a multi-stage complex process of oxidation of iron minerals, particularly magnetite [33] and metallic iron [34]. The WS1 sample heated in an air atmosphere is also characterized by a sharp drop in mass with a peak at 619 °C, which corresponds to graphite oxidation [33]. In the case of the WS2 sample, two peaks are clearly distinguished, which are related to graphite oxidation, and then the resulting carbon dioxide can react with the remaining graphite to form carbon monoxide at 712 °C [35]. However, the second peak more likely corresponds to wustite and magnetite oxidation to hematite in air [36,37]. The heating in the argon atmosphere of both the samples causes the reduction in α-Fe_2_O_3_, which is generated before from α-FeOOH, to magnetite by graphite at the temperatures around 850 °C, then the produced magnetite is reduced to wustite, which is demonstrated by significant endothermic peaks close to 1000 °C [38].

#### 3.1.4. Analysis of Iron Forms

**Figure 5:**
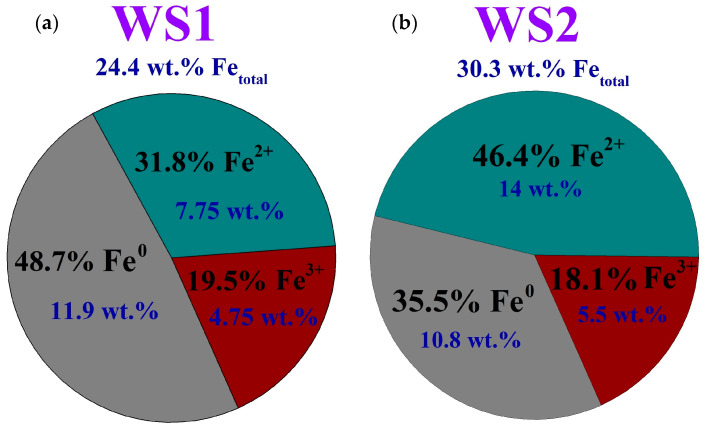
The iron form distribution in the WS1 (**a**) and WS2 (**b**) samples, respectively, according to the potassium dichromate titration methods.

Figure 5 illustrates the distribution of Fe^0^, Fe^2+^ and Fe^3+^ iron in the Waelz slag samples according to the potassium dichromate titration methods.

As reflected by the pie charts, the content of ferric iron in the samples is nearly identical. Almost half of iron is in the metallic form in the WS1 sample, whereas the same value is observed for ferrous iron in the WS2 sample.

**Figure 6:**
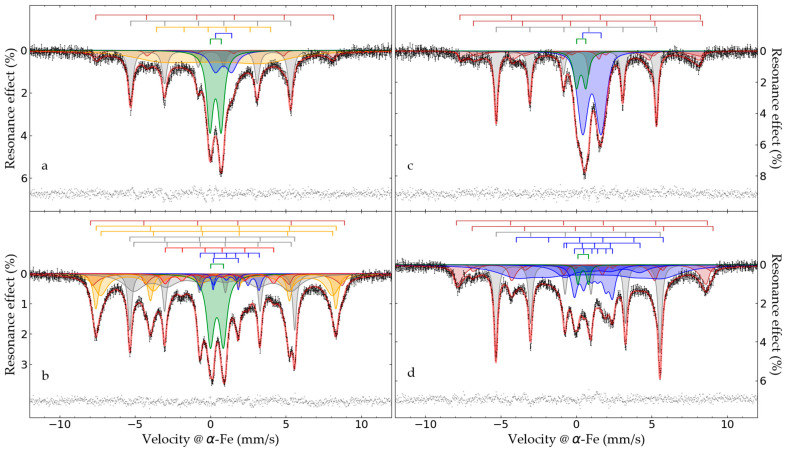
Mössbauer spectra of the WS1 (**a**,**b**) and WS2 (**c**,**d**) samples at 296 (**a**,**c**) and 78 K (**b**,**d**). The subspectra simulating the experimental data are indicated in different colors, and the spectrum of residues is shown below.

In order to elucidate iron compounds in the samples, the Mössbauer spectroscopy method was applied. Figure 6 depicts the obtained Mössbauer spectra, while Table 2 lists their parameters.

Mössbauer spectra of the Waelz slag samples obtained at room temperature have a multiplex profile indicating the complex composition of the samples (Figure 6a,c). In the center of this profile, intense asymmetric doublet lines can be identified. Moreover, for the WS1 sample, this doublet has narrower lines than the doublet of the WS2 sample, which is shifted to the region of high velocities. Furthermore, one can distinguish a fairly intense sextet with relatively narrow resonance lines in the spectra of both samples. At the periphery of the spectra, low-intensity and asymmetric resonance lines are observed, which apparently correspond to the extreme lines of sextets of iron oxide compounds.

The reduction in the temperature of collecting the spectra to the boiling point of nitrogen led to a dramatic change in the Mössbauer spectra of both samples (Figure 6b,d). The intensity of the central paramagnetic doublets was decreased noticeably, while the intensity of the peripheral resonance lines was increased. However, the nature of the changes is individual for each sample. In particular, despite a substantial decrease in the intensity of the paramagnetic part of the WS1 sample spectrum, it is quite reliably distinguished (Figure 6b), while the intensity of the same spectrum part for the WS2 sample is too small to identify it ambiguously (Figure 6d).

In addition, the external resonance lines of the sextet with maximum magnetic splitting in the low-temperature spectrum of the WS1 sample are quite intense and narrow, which allows them to be undoubtedly identified, while in the similar spectrum part of the WS2 sample, the same lines remain low-intensity, wide and noticeably distorted (Figure 6d). All these observations indicate a variety of physical transitions with the temperature change and point out differences in the composition of the Waelz slag samples. The positions of the general area center of the experimental spectra obtained at 296 and 78 K are 0.30 ± 0.03 and 0.45 ± 0.02 mm/s for the WS1 sample, as well as 0.49 ± 0.03 and 0.63 ± 0.02 mm/s for the WS2 sample, respectively. It indicates a higher content of Fe^2+^ compounds in the WS2 sample [39], which is consistent with the XRD (Figure 1 and Figure 2) and titration (Figure 5) analyses.

All experimental spectra can be satisfactorily described by a superposition of 5–7 subspectra depending on the sample (Table 2). For all the spectra, one or two sextets with maximum values of ultrafine magnetic field can be identified (Nos. 1 and 2, Table 2). The profiles of these sextets can be described using the many-state superparamagnetic relaxation model [40,41]. Obviously, these subspectra can be related to solid solutions of magnetite–maghemite Fe_3−δ_O_4_ [42], where δ varies from 0 to 1/3 for magnetite and maghemite, respectively [43]. A sextet with a lower isomer shift and a higher magnetic hyperfine splitting is attributed to Fe^3+^ in the tetrahedral positions of the spinel structure, while a sextet with a higher isomer shift and a lower magnetic hyperfine field is related to Fe^n+^ (2 ≤ n ≤ 3) in the octahedral positions of the spinel structure [44]. Evidently, the WS1 sample contains a more oxidized form of non-stoichiometric Fe_3−δ_O_4_, whereas the WS2 sample contains predominantly reduced iron atoms in octahedral positions, which is confirmed by a high value of the isomeric shift of the second sextet. Based on the data obtained at 78 K and the application of the method described in [45], we determined that the WS1 sample contains Fe_2_._667_O_4_, i.e., pure maghemite γ-Fe_2_O_3_, and the WS2 sample contains Fe_2_._824_O_4_. Assuming that the magnetic anisotropy constants for maghemite and non-stoichiometric magnetite are 4.6 × 10^3^ J/m^3^ [46,47] and 1 × 10^4^ J/m^3^ [48,49], respectively, and using the obtained relaxation parameters according to [50], we estimated the size of magnetic domains for γ-Fe_2_O_3_ as 15.1 ± 0.8 nm and for Fe_2_._824_O_4_ as 13.8 ± 0.3 nm.

In all the spectra for both samples, sextets are also reliably identified (No. 3 at 296 and 5 at 78 K, Table 2), which clearly refer to metallic iron α-Fe [51]. Taking into account the significantly wider resonance lines of the corresponding sextet of the WS2 sample compared to the WS1 sample one, we suggest a lower content of impurities in metallic iron of the WS2 sample. In addition, a sextet with broadened lines is in the low-temperature spectrum of the WS1 sample detected (No. 6 at 78 K in Table 2), which is obviously related to iron alloys with transition metals [52,53,54,55].

Another similar component in all the spectra of both the samples is a paramagnetic doublet (No. 6 at 296 K and No. 10 at 78 K, Table 2), which corresponds to Fe^3+^ in an octahedral environment [39]. As the temperature of spectra collecting decreased, the intensity of this doublet decreased by one-third for the WS1 sample, while for the WS2 sample, it was reduced to almost one-fourth of its original intensity. Obviously, compounds with magnetic ordering make a major contribution to this paramagnetic part of the WS2 sample spectrum at 78 K, which is discussed below. The WS1 sample has a significant part of a paramagnetic compound at both temperatures, which is likely lepidocrocite γ-FeOOH [56,57].

The Mössbauer spectra obtained at 296 K of both the samples also contain other doublets with wide resonance lines (No. 5 at 296 K, Table 2), which are attributed to Fe^2+^ in tetrahedral and octahedral oxygen environments [39]. These subspectra substantially vary in intensity, isomer shift and quadrupole splitting. When the WS2 sample is cooled to 78 K, this doublet is completely transformed into sets of sextets with high values of the isomer shift and the magnetic hyperfine field strength in the range from 78 to 302 kOe (Nos. 7–9 at 78 K for WS2, Table 2). Obviously, these spectra components, as well as partially the described above paramagnetic doublet Fe^3+^, are related to non-stoichiometric iron monoxide Fe_1−x_O [58,59]. The temperature decreasing to 78 K causes Fe_1−x_O transition to an antiferromagnetic state [60,61], which corresponds to the spectrum of a complex profile with magnetic splitting [62] and is described for these samples by either one or three sextets (Figure 6). The low-temperature spectrum of the WS2 sample contains the derivatives of the paramagnetic doublet Fe^2+^, including a low-intensity doublet with narrow resonance lines (No. 9 at 78 K, Table 2), as well as a sextet with a low magnetic hyperfine field (No. 8 at 78 K, Table 2). The set of hyperfine parameters of this component is mismatched with the known binary iron compounds, which can be formed in our study.

In addition, the spectrum of the WS1 sample at 296 K contains a sextet with strongly broadened resonance lines (No. 4, Table 2). This broadened sextet, along with a part of the paramagnetic doublet Fe^3+^, obviously belongs to goethite. Indeed, when the temperature decreases to 78 K, these components are transformed into reliably fixed sextets (Nos. 3 and 4 at 78 for WS1, Table 2) with hyperfine parameters (particularly, the quadrupole shift) of goethite [63,64].

Another unique component of the WS1 sample is observed in low-temperature spectra (No. 7 at 78 K for WS1, Table 2), which can be attributed to iron carbide—Θ-Fe_3_C [65,66].

#### 3.1.5. Zinc and Copper Phase Analysis

**Figure 7:**
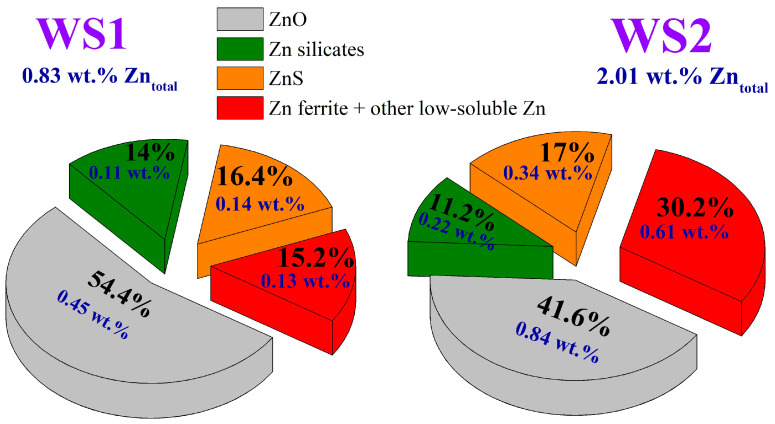
Phase composition of zinc minerals in the Waelz slag samples.

**Figure 8:**
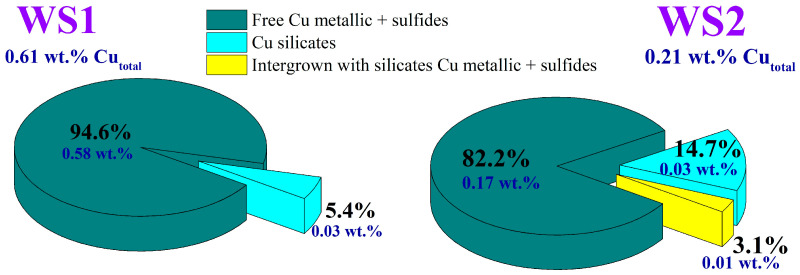
Phase composition of copper minerals in the Waelz slag samples.

Figure 7 and Figure 8 demonstrate the distribution of zinc and copper compounds in the Waelz slag samples according to the corresponding chemical phase analysis methods.

As shown in Figure 7, the major zinc phase of the WS1 and WS2 samples is free zinc oxide. There are also substantial amounts of zinc sulfide, silicates and ferrite. The presence of zinc ferrite is compatible with the Mössbauer spectra, where it can be included in the paramagnetic doublet (No. 6 at 296 K and No. 7 at 78 K, Table 2). For example, according to our estimates, the maximum possible relative area of a zinc ferrite subspectra in both the WS2 samples is 2.9%, which is substantially higher than the values and can be calculated from the zinc contents in Figure 6. The distribution of zinc forms in the samples is quite similar, but a difference in the ratio of zinc oxide and zinc ferrite is in evidence. The found zinc phases are consistent with the study [67], where the Waelz slag samples were also characterized.

As reflected in Figure 8, copper is mostly present as a sum of metallic and sulfide forms in both samples. In the WS1 sample, almost all copper is concentrated in the free metallic and sulfide forms; silicates account for slightly more than 5%. In the WS2 sample, the content of copper silicates is about 15%. It should be noted that about 3% of metallic and sulfide copper is intergrown with silicates. However, the same as in the WS1 sample, the main part of copper in the WS2 sample is in free metal and sulfide.

**Figure 9:**
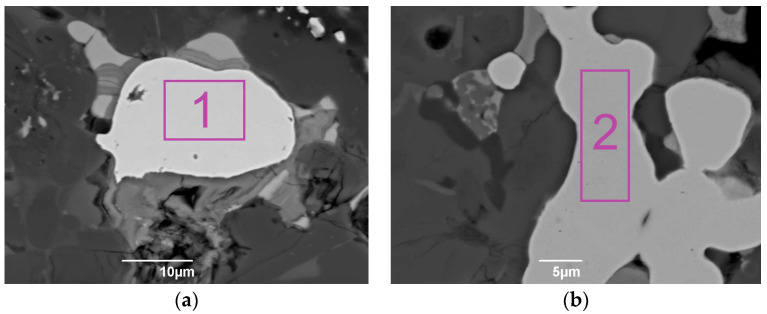
SEM photomicrographs of typical metallic iron particles of the WS1 (**a**) and WS2 (**b**) samples, where copper was detected.

In order to clarify copper mineralogical distribution, we used SEM with electron microprobe analysis. Figure 9 demonstrates typical sites of the WS1 and WS2 samples with increased copper content. Table 3 shows the chemical composition of the areas of Figure 9.

It has been found that metallic iron contains an increased amount of metallic copper, which can also be one of the reasons for the wide resonance lines of α-Fe in the Mössbauer spectra (Figure 6). The results of the SEM-EDX analysis showed that all copper was present in the metallic form with metallic iron, and copper sulfides were not detected. This leads to the assumption that metallic copper is the main copper-containing phase of the Waelz slag samples.

#### 3.1.6. Sieve Analysis

Table 4 shows the yield of fractions of the Waelz slag samples after the sieve analysis and the contents of Fe, Zn and Cu in the fractions.

As can be seen from the results of the sieve analysis, it is difficult to determine a corresponding correlation of copper and zinc content with the fraction size. A clear dependence is a decrease in the amount of iron content with a reduction in the fraction size. In addition, it should be noted that there is a significant concentration of zinc in fine fractions of the WS1 sample, especially in −0.1 mm, which can be considered for recycling in the Waelz process. However, the yield of this fraction is too small to influence the holistic processing technology of the Waelz slag. Therefore, the sieve analysis showed no significant segregation of valuable elements in any fraction, so a further magnetic separation study was carried out for the entire Waelz slag samples.

### 3.2. Magnetic Separation

#### 3.2.1. WS1 Sample Separation

**Figure 10:**
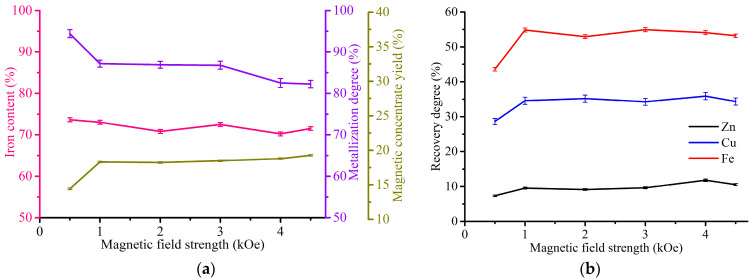
Indexes of magnetic separation of WS1 sample (94.6% −0.054 mm): (**a**) the effect of magnetic field strength on the Fe content in the magnetic concentrate, Fe metallization degree of the concentrate and the yield of the concentrate; (**b**) the effect of magnetic field strength on the recovery degree of iron, copper and zinc.

Figure 10 shows the effect of magnetic field strength on the magnetic separation indexes for the WS1 sample, 94.6% of which was ground to −0.054 mm.

As can be seen from the results obtained, an initial increase in the magnetic field strength from 0.5 kOe to 1 kOe led to a sharp increase in the recovery degree of iron from 43.6 to 54.8%, zinc from 7.3 to 9.5% and copper from 28.6 to 34.6%, as well as the yield of magnetic concentrate from 14.4 to 18.3%, while a sharp decrease in the iron metallization degree from 94.4 to 87.2% also occurred. A further increase in the magnetic field strength to 4.5 kOe has an insignificant effect on the process characteristics. The iron content variation range is 70.2–73.6%, which corresponds to an iron-rich concentrate [68]. The best results were obtained for 1 kOe, so we fixed this value for further investigation of the WS1 sample treatment.

**Figure 11:**
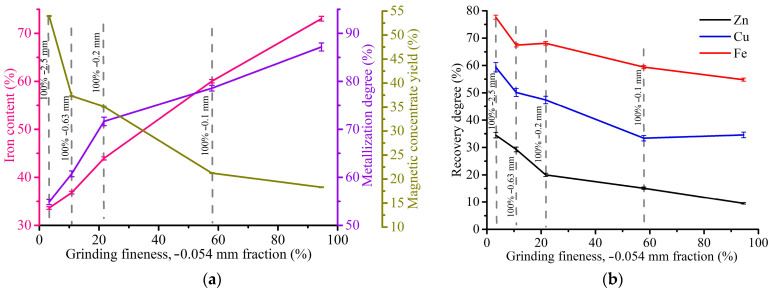
Indexes of magnetic separation of the WS1 sample at 1 kOe: (**a**) the effect of grinding fineness on the Fe content in the magnetic concentrate, Fe metallization degree of the concentrate and the yield of the concentrate; (**b**) the effect of grinding fineness on the recovery degree of iron, copper and zinc.

Figure 11 demonstrates the effect of grinding fineness on the magnetic separation indexes for the WS1 sample at a magnetic field strength of 1 kOe.

As can be seen from the presented graphs, an increase in grinding fineness from −2.5 mm to −0.054 mm resulted in a considerable rise of the iron concentrate grade from 33.6 to 73%, and the degree of its metallization from 54.9 to 87.2%. On the other hand, there is a drop in the yield of magnetic concentrate from 54 to 18.3% and the recovery degree of iron from 77.6 to 54.8%, zinc from 34.5 to 9.5% and copper from 59.4 to 34.6%. In this case, it is important to grind the WS1 sample as fine as possible to obtain high-grade iron concentrate.

**Figure 12:**
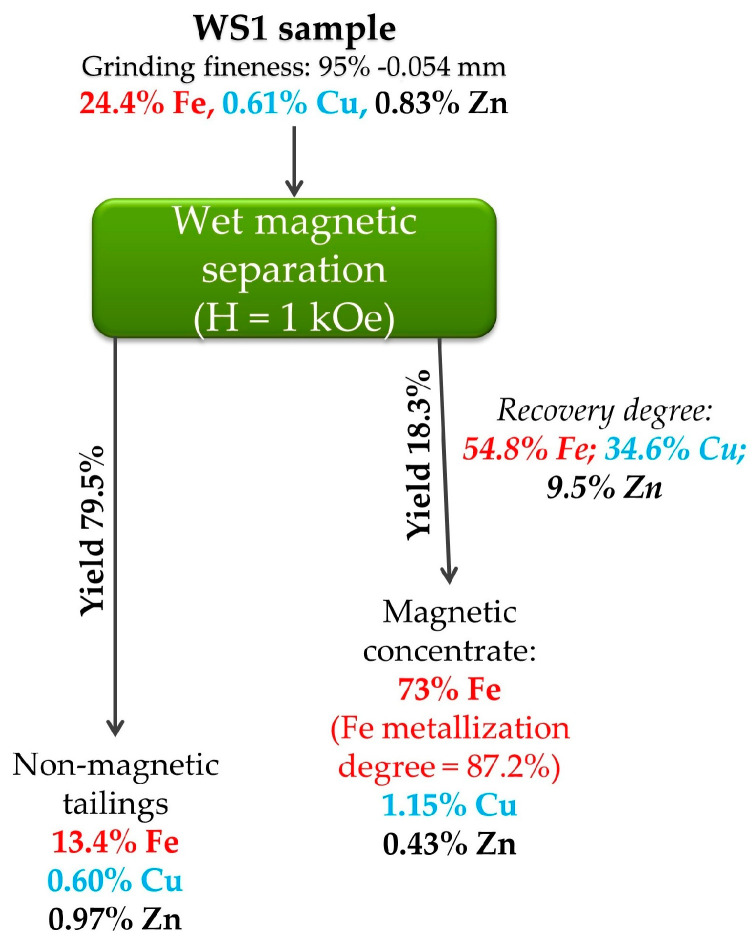
The best results of magnetic separation for the WS1 sample.

Figure 12 starkly illustrates the best results of magnetic separation for the WS1 sample. 

Preliminary grinding of the sample to 0.054 mm before wet magnetic separation at a magnetic field strength of 1 kOe allows for obtaining a magnetic concentrate containing 73% iron. It is noteworthy that the copper content of 1.15% in the concentrate is very attractive for its extraction, although the recovery degree of Cu in the concentrate is only slightly more than 30%. Moreover, zinc content in the concentrate remains increased to recycle it in ironmaking. The mineralogical composition of zinc phases (Figure 7) confirmed its presence in non-magnetic phases without possible connection with metallic iron, so the residual Zn content in the concentrate is likely due to mechanical impregnation. Thus, there is no complete segregation and concentration of valuable elements using magnetic separation.

Table 5 lists the chemical composition and recovery degree of various elements in the best concentrate derived from the WS1 sample.

The data indicate that Ni, As and Sb are substantially passed in the concentrate along with Fe. The amounts of Fe, Ni, As and Sb in the concentrate increased approximately threefold compared with the WS1 sample. The main part of Zn, Pb and Mn passed in the tailings. The recovery rate of Cr and P of about 22–24% and their increased contents compared with the WS1 sample are evidence of partial presence along with metallic iron that was shown previously by SEM (Figure 9a, Table 3).

**Figure 13:**
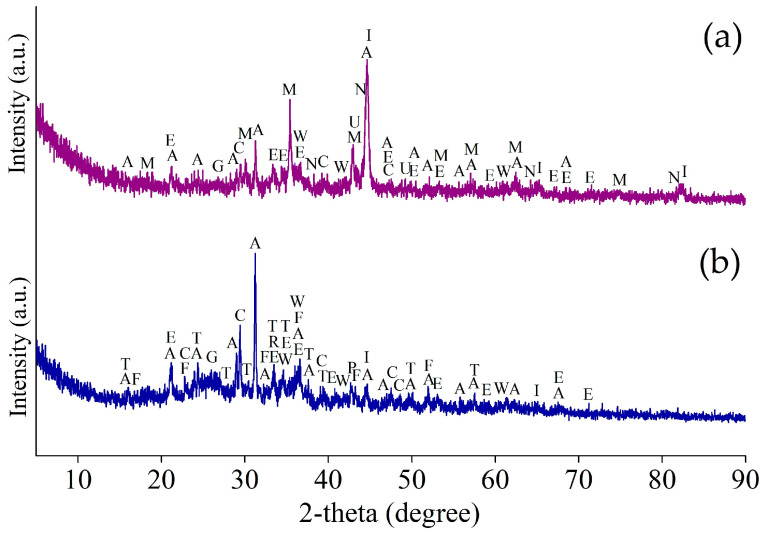
The XRD pattern of the magnetic concentrate (**a**) and the tailings (**b**) obtained by magnetic separation of WS1 sample at 1 kOe and −0.054 mm grinding fineness (95%); I—iron α-Fe (96-900-8537); M—maghemite γ-Fe_2_O_3_ (96-901-2693); W—wustite Fe_1−x_O (96-210-6937); E—goethite α-FeOOH (96-100-8768); C—calcite CaCO_3_ (96-702-0140); A—akermanite Ca_2_Al_0_._99_Mg_0_._46_Si_1_._52_O_7_ (96-900-6116); G—graphite C (96-900-8570); U—metallic copper Cu (96-901-3019); N—magnesiowuestite Fe_0_._4_Mg_0_._6_O (96-900-6066); P—periclase MgO (96-500-0226); F—forsterite Mg_2_SiO_4_ (96-900-0268); T—monticellite CaMgSiO_4_ (96-210-1345); R—merwinite Ca_3_MgSi_2_O_8_ (96-156-0349).

Figure 13 shows the XRD patterns of the magnetic concentrate and the tailings obtained in the optimal conditions of the magnetic separation of the WS1 sample.

Most of the base elements in the magnetic concentrate and tailings are in the same minerals as in the WS1 sample. The main parts of metallic iron and magnetite are predictably in the concentrate, whereas the majority of goethite and wustite are in the tailings. Metallic copper is also identified in the concentrate. Iron-free minerals mostly remain in the tailings.

#### 3.2.2. WS2 Sample Separation

**Figure 14:**
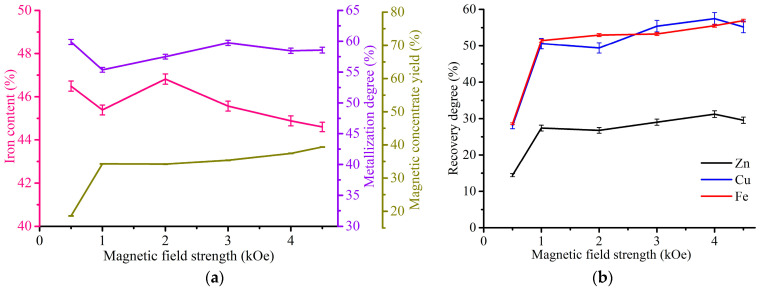
Indexes of magnetic separation of WS2 sample: (**a**) the effect of magnetic field strength on the Fe content in the magnetic concentrate, Fe metallization degree of the concentrate and the yield of the concentrate; (**b**) the effect of magnetic field strength on the recovery degree of iron, copper and zinc.

Figure 14 depicts the change in the magnetic separation indexes depending on the magnetic field strength for the WS2 sample ground to −0.054 mm.

In the same way as the WS1 sample, increasing the magnetic field strength from 0.5 to 1 kOe caused a sharp rise in the recovery degree of iron, zinc and copper, as well as the yield of magnetic concentrate. However, the values of the increased indexes are dissimilar to the WS1 sample, especially the concentrate yield. The rise from 0.5 to 1 kOe led to an increment of the recovery degrees of zinc from 14.4 to 27.4%, copper from 28 to 50.6% and iron from 28.6 to 51.4%, as well as the yield of the concentrate from 18.6 to 34.3%. The iron content and its metallization degree are in the ranges of 44.6–46.8% and 55.4–59.9%, respectively, which is significantly lower than in the concentrates from the WS1 sample. The chosen optimal conditions for further study were a magnetic field strength of 2 kOe for the best iron grade of the concentrate.

**Figure 15:**
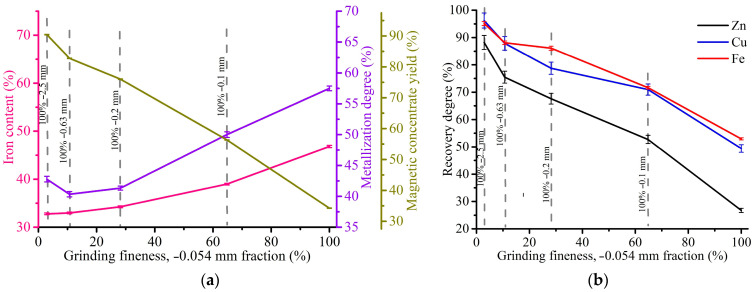
Indexes of magnetic separation of WS2 sample: (**a**) the effect of grinding fineness on the Fe content in the magnetic concentrate, Fe metallization degree of the concentrate and the yield of the concentrate; (**b**) the effect of grinding fineness on the recovery degree of iron, copper and zinc.

Figure 15 demonstrates the effect of grinding fineness on the magnetic separation indexes at 2 kOe for the WS2 sample.

As in the case of the WS1 sample, an increase in grinding fineness from −2.5 mm to −0.054 mm led to a decrease in the yield of magnetic concentrate from 90.4 to 34.2% and the recovery degrees of iron from 95.1 to 51.9%, zinc from 88.2 to 26.8% and copper from 96.2 to 49.4%. Therefore, the results show that the magnetic separation is inefficient for rough ground of the WS2 sample due to insignificant separation degree and a low content of iron in the concentrates. The iron contents are in the range of 32.8–46.8%. Only grinding fineness −0.054 mm gives the iron grade more than 40%, but it is still substantially lower than the best concentrate derived from the WS1 sample (Figure 12).

**Figure 16:**
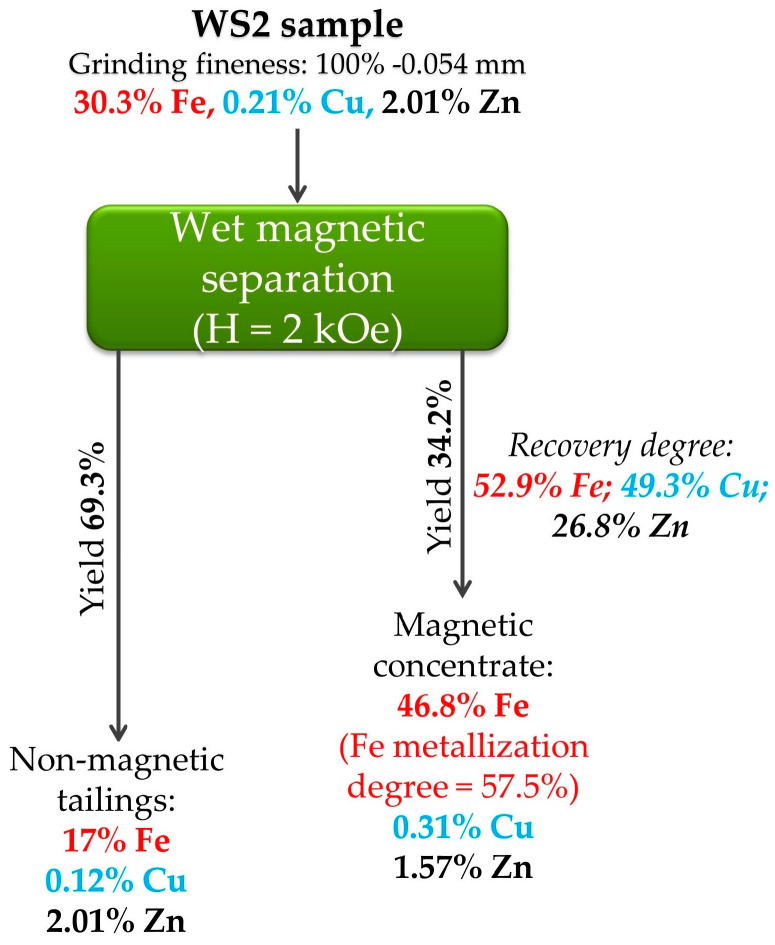
The best results of magnetic separation for the WS2 sample.

Figure 16 illustrates the best results of magnetic separation for the WS2 sample. Preliminary grinding of the WS2 sample to −0.054 mm before wet magnetic separation at a magnetic field strength of 2 kOe provides obtaining a magnetic concentrate containing 46.8% Fe. It can be seen that although the iron recovery degree after magnetic separation of the WS1 and WS2 samples are almost similar, the grade of magnetic concentrate derived from the WS2 sample is far lower, and the iron content in the tailings derived from the WS2 sample is higher. As for the WS1 sample, the zinc content is too high for ironmaking in the concentrate obtained from the WS2 sample.

Table 6 shows the chemical composition and degree of recovery of various elements in the best concentrate obtained from the WS2 sample. Contrary to the lower iron content compared with the best WS1 concentrate (Figure 12), the WS2 concentrate seems to be more attractive for recycling due to the low contents of As, Sb and P. The recovery degrees of Pb and Cr in the WS2 concentrate are consistent with the WS1 concentrate, while the recovery degrees of Cu, Zn, Mn, Ni and As are substantially higher, which is due to the higher yield of the concentrate.

**Figure 17:**
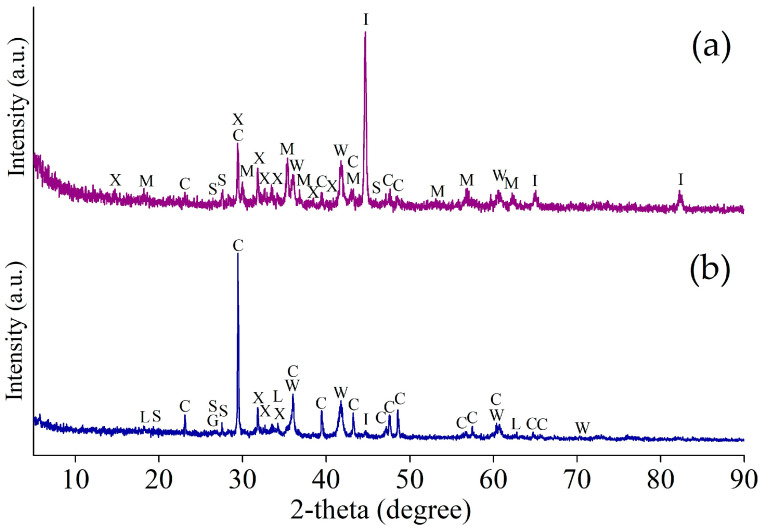
The XRD pattern of the magnetic concentrate (**a**) and the tailings (**b**) obtained by magnetic separation of WS2 sample at 1 kOe and −0.054 mm grinding fineness (95%); I—iron α-Fe (96-411-3937); M—magnetite Fe_3−δ_O_4_ (96-900-9769); W—wustite Fe_1−x_O (96-900-9767); C—calcite CaCO_3_ (96-901-6707); G—graphite C (96-900-0047); X—tricalcium silicate Ca_3_SiO_5_ (96-901-5422); S—pseudowollastonite CaSiO_3_ (96-900-2180); L—portlandite Ca(OH)_2_ (96-100-0046).

Figure 17 shows the XRD pattern of the magnetic concentrate and the tailings obtained in the optimal conditions of the magnetic separation of the WS2 sample.

As in the case of the WS1 sample, the main part of iron in the magnetic concentrate was retained as phases of the original WS2 sample. Calcium-containing phases such as carbonate, hydroxide and silicates are mostly found in non-magnetic tailings.

## 4. Discussion

The characterization study of the Waelz slag has shown a significant difference in the chemical and mineralogical compositions of the Waelz slag samples from different plants. The WS1 sample contains substantial percentages of copper and arsenic, which are unusual for EAFD processing. This fact is likely due to a variety of charge materials for the Waelz process at the Chelyabinsk zinc plant, where the WS1 sample was generated. In addition to the EAFD [69], different raw materials are treated in the Waelz kilns of this plant, such as zinc leaching residue [70], copper smelter dust [71] and others. A significant part of the elements contributed by the raw materials that were previously processed in the kiln can be present as a trace in the charge for a long time. It is notable that the increased contents of arsenic and copper in the WS1 sample are not an isolated case of the composition of the Waelz slag from the EAFD processing. For example, the paper [17] includes the composition with 0.089% As, while the studies [67,72] deal with the Waelz slag samples of 0.75 and 1.3% Cu, respectively. On the one hand, the presence of harmful arsenic essentially complicates the recycling of the WS1 sample. On the other hand, a high content of copper is attractive for its recovery. The WS2 sample composition is typical for the EAFD processing because, in contrast to the plant of the generation of the WS1 sample, “Alfatrans” LLC processes only the EAFD.

**Figure 18:**
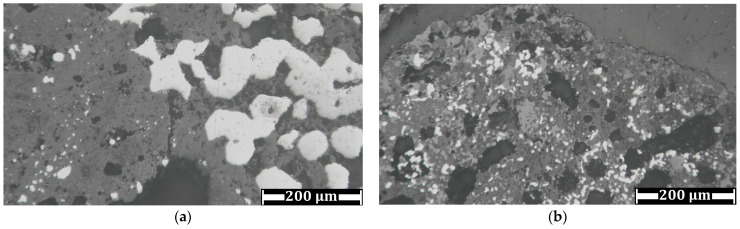
Microstructure of WS1 (**a**) and WS2 (**b**) samples.

Based on the chemical composition of the samples (Table 1), one might estimate a difference in the conditions of the Waelz process used at the plants. The WS1 sample has a higher carbon content and a lower residual zinc content than the WS2 sample, which probably indicates a higher processing temperature. An important target of the Waelz process is to prevent substantial charge smelting, thereby avoiding skull formation. The addition of an excess of coke breeze is one of the possible solutions to avoid skull formation at elevated temperatures [73]. Although it results in a higher consumption of coke breeze, the zinc recovery into the Waelz oxide increases, and the zinc content in the Waelz slag decreases. Conversely, if there is no excess of coke breeze, elevated temperatures have an adverse effect on the charge processing. In this case, as can be seen from the chemical composition of the WS2 sample (Table 1), there is a higher residual zinc percentage, as well as a considerable calcium content, which is due to the addition of a higher amount of calcium-containing fluxes to prevent charge smelting. Figure 18 shows the photomicrographs of the samples, which clearly demonstrates the difference in the conditions of the Waelz slag generation.

As obviously shown from the photomicrographs, the average size of metallic iron particles in the WS1 sample is significantly larger than the WS2 sample. Since the crucial factor of the efficiency of the magnetic separation process is the size of iron particles [74], this is the main reason for the difference in the results between the WS1 (Figure 10, Figure 11 and Figure 12) and WS2 (Figure 14, Figure 15 and Figure 16) samples. Undoubtedly, various compositions and microstructures of the samples from different plants complicate the development of a universal technology for Waelz slag recycling.

The magnetic separation led to insufficient segregation of iron, zinc and copper in the corresponding products. The recovery degree of iron into the magnetic concentrate in the range of 52.9–54.8% at the best conditions (Table 5 and Table 6) is too low, and the residual amount of iron in the tailings of 13.4–17% (Figure 12 and Figure 16) is too high to consider the separation successful. This is due to the presence of significant amounts of low-magnetic wustite Fe_1−x_O, goethite α-FeOOH and lepidocrocite γ-FeOOH (Figure 1 and Figure 2, Table 2), as well as the above-mentioned small-sized metallic iron particles impregnated in various non-magnetic phases. Moreover, as shown by our preliminary experiments, the presence of the low-magnetic phases leads to a significant retaining iron in the tailings even using high-gradient magnetic separation at a magnetic field strength of 8–16 kOe. Certainly, the formation of goethite and lepidocrocite is improbable during high-temperature treatment in the Waelz kiln, but they are likely the main corrosion products of metallic iron [75,76,77] during storage of the Waelz slag in stockpiles or dumps. Therefore, in the case of magnetic separation, it is important to process the slag immediately after its formation in the kiln.

Since copper is associated with metallic Fe as non-magnetic metallic Cu (Figure 8 and Figure 9), the grinding process leads to a partial detachment of copper from small iron grains and subsequent decreasing copper recovery into the magnetic concentrate. The association of iron and copper agrees well with our experimental data, especially for the WS2 sample (Figure 14 and Figure 15), where curves of Cu recovery resemble curves of Fe recovery. Regarding zinc, its residual contents in the concentrate are quite high, as in the study [23], which is likely due to a substantial part of zinc ferrite being associated with magnetite [78]. Thus, the selective segregation of copper and zinc in the magnetic or non-magnetic fraction, respectively, was not achieved using magnetic separation. However, on the positive side, the metalized iron concentrate was obtained from the WS1 sample with a high copper content that was attractive for its recovery by hydrometallurgical methods of copper production [79].

This study has potential limitations in the number of research objects. We investigated the behavior of the valuable elements during the magnetic separation using only two different Waelz slag samples. A variety of the used charge materials in the Waelz process, as well as different technological kiln parameters, can result in a wide range of the Waelz slag compositions that consequently influence the grade of the magnetic separation products and the recovery of valuable elements. Therefore, it is further important to study the effect of the Waelz process parameters and the processed EAFD composition on the composition of the produced Waelz slag and the efficiency of its subsequent magnetic separation, which can identify the optimal conditions for the recovery of valuable elements and obtain the high-grade recyclable products.

One of the efficient solutions to improve the results of magnetic separation is changing the parameters of the Waelz process to reduce as much iron as possible. The well-known practice is the reduction and subsequent reoxidation of iron at the end of the kiln using air blow to induce exothermic reactions and, as a result, decrease the consumption of coke and natural gas [80]. However, in terms of iron recovery by magnetic separation, this practice is unfavorable, so preventing iron oxidation should be applied. In principle, not only Waelz oxide but also Waelz slag should be considered a product of the process rather than as a waste to obtain its optimal composition for subsequent recycling.

## 5. Conclusions

The characterization study of the Waelz slag has shown the detailed chemical and phase composition of the samples from two different plants. Touching upon the mineralogical composition of the Waelz slag samples, several points can be summarized:

- Iron is mainly present in the samples as metallic Fe, Fe_1−x_O, Fe_3−δ_O_4_ and FeOOH;
- Zinc was discovered in the form of oxide, silicates, sulfides and ferrite;
- Copper was detected only in metallic form associated with metallic iron.

It was found that there is a substantial variety of the chemical and mineralogical compositions of the Waelz slag samples from the different plants. The characterization study afforded a basis for clarification of the behavior of elements during the magnetic separation.

Magnetic separation was found as a way to derive the Fe-rich product from the Waelz slag. The magnetic separation at the best conditions led to the following results:

- The concentrate with 73% Fe, 0.43% Zn and 1.15% Cu was obtained from the WS1 sample containing 24.4% Fe, 0.83% Zn and 0.61% Cu at grinding fineness of −0.054 mm (94.6%) and magnetic field strength of 1 kOe;
- The concentrate with 46.8% Fe, 1.58% Zn and 0.31% Cu was derived from the WS2 sample containing 30.3% Fe, 2.01% Zn and 0.21% Cu at grinding fineness of −0.054 mm and magnetic field strength of 2 kOe.

The chemical and mineralogical composition, as well as microstructure, especially the grain size of metallic iron, have a significant effect on the results and efficiency of the magnetic separation.

## Figures and Tables

**Table 1:** Chemical composition of the Waelz slag samples, wt.%.

Sample	Fe	Zn	Cu	Ca	Si	Al	Mn	Cr	Na	K	Ti	P	Ni	Pb	As	Sb	V	S	C
WS1	24.4	0.83	0.61	9.92	5.61	1.75	2.25	0.32	0.49	0.27	0.140	0.17	0.046	0.27	0.22	0.041	0.010	1.84	19.1
WS2	30.3	2.01	0.21	20.0	4.70	1.08	2.87	0.31	0.98	0.38	0.084	0.18	0.025	0.38	0.003	0.059	0.015	1.39	5.1

**Table 2:** Hyperfine parameters ^1^ for the depicted in Figure 6 Mössbauer spectra of the WS1 and WS2 samples.

T ^2^	No.	WS1 Sample						WS2 Sample					
		Phase	δ	ε {Δ = 2ε}	Г_exp_	H_eff_	S	Phase	δ	ε {Δ = 2ε}	Г_exp_	H_eff_	S
K			mm/s	mm/s	mm/s	kOe	%		mm/s	mm/s	mm/s	kOe	%
296	1	γ-Fe_2_O_3_	0.29(2)	−0.02(2)	0.37(6)	489(2)	4.8(5)	Fe_2_._824_O_4_	0.28(1)	−0.03(1)	0.23(3)	494(1)	12(1)
	2								0.79(3)	−0.03(3)	0.35(9)	471(2)	7(1)
	3	α-Fe	0.02(1)	−0.01(1)	0.38(1)	328.8(2)	23.7(7)	α-Fe	0.00(1)	0.00(1)	0.28(1)	329.4(1)	29.0(4)
	4	α-FeOOH	0.31(5)	−0.12(4)	4.1(3)	235(6)	36(2)						
	5	Fe^2+^_Td_	0.86(2)	{1.08(2)}	0.88(6)		11.0(9)	Fe^2+^_Oh_	1.04(1)	{1.21(1)}	0.82(1)		40.3(6)
	6	Fe^3+^_Oh_	0.33(1)	{0.74(1)}	0.51(1)		24.2(6)	Fe^3+^_Oh_	0.32(1)	{0.62(1)}	0.46(2)		11.4(3)
78	1	γ-Fe_2_O_3_	0.45(1)	0.00(1)	0.28(1)	522(3)	7(1)	Fe_2.824_O_4_	0.41(1)	−0.05(1)	0.40(2)	515.2(8)	12.9(5)
	2								1.12(2)	−0.05(1)	0.31(5)	494(1)	3.7(4)
	3	α-FeOOH	0.49(1)	−0.13(1)	0.41(3)	493.2(3)	13(2)						
	4		0.54(1)	−0.13(1)	1.08(5)	476(2)	20(2)						
	5	α-Fe	0.12(1)	0.00(1)	0.30(1)	338.9(1)	13.5(6)	α-Fe	0.11(1)	0.00(1)	0.28(1)	337.1(1)	25.4(4)
	6	Fe alloy	0.13(2)	0.00(1)	1.6(1)	324(2)	21(1)						
	7	Θ-Fe_3_C	0.39(2)	0.20(1)	0.54(4)	223(1)	4.3(4)	Fe_1−x_O	0.93(4)	−0.04(1)	3.0(2)	302(3)	31(2)
	8	Fe^2+^_Oh_	1.31(1)	−0.04(1)	0.36(2)	120.9(5)	4.6(2)	Fe_1−x_O	1.24(4)	0.45(6)	1.4(1)	156(3)	10(1)
	9	Fe^2+^_Td_	1.01(1)	{1.67(1)}	0.21(2)		1.4(1)	Fe_1−x_O	1.18(1)	−0.04(1)	0.46(2)	78.0(4)	13.8(4)
	10	Fe^3+^_Oh_	0.42(1)	{0.86(1)}	0.60(1)		16.3(2)	Fe^3+^_Oh_	0.46(1)	{0.70(1)}	0.30(2)		2.9(2)

^1^ δ—isomer shift relative to α-Fe at 296 K; ε {Δ = 2ε}—quadrupole shift {splitting}; Γ_exp_—experimental line width; H_eff_—magnetic hyperfine field; S—relative area. Values in parentheses correspond to error estimates in the last digit; ^2^ T—temperature of spectra collecting.

**Table 3:** Composition of the areas 1 and 2 from the photomicrographs of Figure 9, wt.%.

No.	Area	Composition, wt.%		
		Fe	Cr	Cu
1	Metallic iron	98.12	0.61	1.17
2	Metallic iron	99.20	-	0.80

**Table 4:** The yield of the fractions and content of Fe, Zn and Cu in the Waelz slag samples.

Fraction, mm	WS1 Sample				WS2 Sample			
	Yield, %	Content, wt.%			Yield, %	Content, wt.%		
		Fe	Zn	Cu		Fe	Zn	Cu
+10	12.04	31.34	0.41	0.56	9.27	30.4	3.05	0.19
−10 + 7	9.71	27.04	0.68	0.71	8.10	30	4.80	0.19
−7 + 2.5	29.77	24.19	0.43	0.71	22.11	31.7	2.39	0.2
−2.5 + 1.6	13.55	23.86	0.43	0.77	12.81	32.3	1.61	0.2
−1.6 + 1	15.39	24.22	0.51	0.77	18.38	31.9	1.62	0.19
−1 + 0.4	12.59	24.77	0.61	0.81	18.57	30.6	1.79	0.19
−0.4 + 0.2	3.28	20.91	1.06	0.80	6.61	30	1.9	0.2
−0.2 + 0.1	2.15	18.46	2.33	0.77	2.42	29.5	2.31	0.22
−0.1	1.53	15.93	4.8	0.80	1.74	26	3.16	0.3
Total	100	25.0	0.61	0.73	100	31.1	2.27	0.20

**Table 5:** Chemical composition of the magnetic concentrate obtained from the WS1 sample at 1 kOe after grinding of 94.6% particles to −0.054 mm.

Element	Fe	Cu	Zn	Pb	Cr	Mn	Ni	P	As	Sb
Content, wt.%	73.0	1.15	0.43	0.12	0.42	0.87	0.16	0.21	0.64	0.13
Recovery degree, %	54.8	34.6	9.5	8.3	24.1	7.1	61.7	22.7	53.4	59.3

**Table 6:** The chemical composition of the magnetic concentrate obtained from the WS2 sample at 2 kOe after grinding to −0.054 mm.

Element	Fe	Cu	Zn	Pb	Cr	Mn	Ni	P	As	Sb
Content, wt.%	46.8	0.31	1.57	0.087	0.21	1.59	0.057	0.08	0.015	0.037
Recovery degree, %	52.9	49.3	26.8	7.8	23.5	19	77.5	15.2	100	21.2

**Figure A1:**
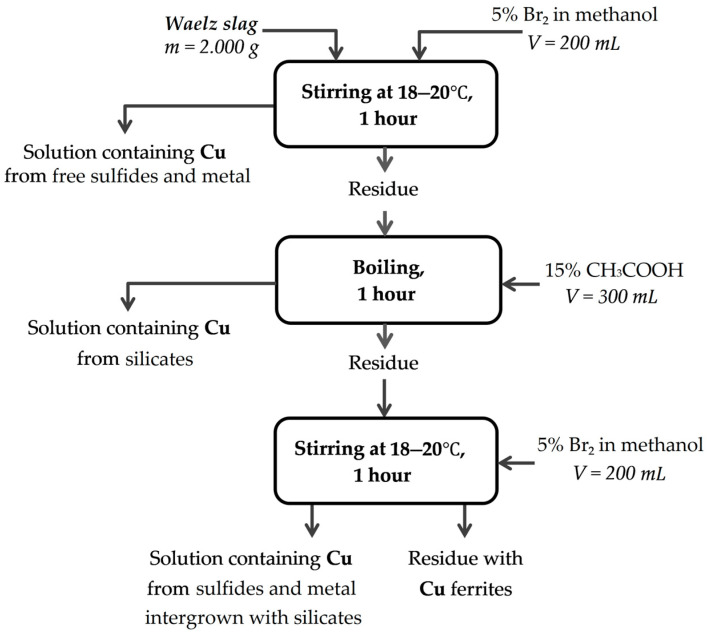
Method of selective dissolution of copper minerals.

## Data Availability

The data presented in this study are available on request from the corresponding author due to privacy reasons.

## Appendix A

Figure A1 demonstrates the steps of selective dissolution of copper minerals used for the WS1 and WS2 samples.
